# Ability of different edible fungi to degrade crop straw

**DOI:** 10.1186/s13568-018-0731-z

**Published:** 2019-01-07

**Authors:** Liang Huang, Ning Sun, Litong Ban, Yu Wang, Hongpeng Yang

**Affiliations:** College of Agronomy and Resources Environment, Tianjin Agricultural University, Tianjin, 300384 China

**Keywords:** Edible fungi, Crop straw, Lignocellulolytic enzymes

## Abstract

Extracellular enzymes play an important role in the growth and development of edible fungi. Extracellular enzyme activities have also become an important object of measurement. In this study, *Agaricus brunnescens* Peck, *Coprinus comatus*, and *Pleurotus ostreatus* were compared in terms of their enzyme production in liquid-and solid-state fermentation. Differences in the ability of various types of edible fungi to utilize biomass raw materials were analyzed by monitoring the fiber degradation rate during crop straw degradation, and changes in their cellulolytic enzyme systems during growth and metabolism were discussed. This study provided insights into the changes in the lignocellulose degradation ability of edible fungi during their growth and facilitated the discovery of new approaches to accelerate their growth in culture.

## Introduction

Edible fungi have been appreciated for their flavor and texture and recognized as a nutritious food and important source of bioactive compounds with medicinal value (Cheung et al. 2003). Certain fungal species have been used for their medicinal properties for over 2000 years, and their bioactive compounds that act as immunomodulatory and exhibit anticancer activities have been isolated (Sadler 2010). Since the 1960s, studies have explored the chemical constituents and pharmacological effects of edible fungi and confirmed the special biological effects and medicinal values of edible fungi at a molecular level. For example, polysaccharides and proteoglycans in edible fungi play important roles in biological activities (Laatsch 1992; Mei and Zhang 2007; Ye et al. 2011).

With current scientific focus on functional foods and functional ingredients, the contribution of edible fungi to this category of foods has been investigated. Edible fungi are cultured in media that usually consist of sawdust, cottonseed husk, wheat bran, and other substances. They also contain lignocelluloses, such as cellulose, hemicellulose, and lignin, which can induce edible fungi to secrete cellulose, hemicellulose, and other extracellular hydrolases (Huai-Liang 2010; Lechner and Papinutti 2006; Rani et al. 2008). Lignocellulolytic enzymes are carbohydrate-active enzymes that perform important roles in the carbohydrate metabolism of organisms. Lignocellulolytic enzyme secretion during the fermentation of edible fungi is a necessary physiological function for the transformation of lignocellulolytic matrix during fungal growth (Elisashvili et al. 2002). Therefore, the decomposition degree and efficiency of edible fungi can be understood by detecting the extracellular enzyme activities of edible fungi under different conditions to provide a rapid and enhanced environment for hyphal and sporophore growth. We could understand the trend of changes in the extracellular enzyme activities of edible fungi from the aspect of edible fungal growth. As such, we can further explore the pharmacological activity of edible fungi and improve cultivation techniques.

Crop straw is difficult to be used as an industrial raw material and feed because the cell wall of a plant stem contains lignocellulose (Parisi 1989; Schimpf et al. 2013). Lignocellulose is one of the most widely distributed organic compounds worldwide and has become a limiting factor in the global carbon cycle because of its low degradation efficiency. Traditional lignocellulosic biomass treatment processes include biomass pretreatment, pretreated biomass hydrolysis, fermentation, and biofuel recovery (Muktham et al. 2016). These operations are complex and strict, and the production and purification of lignocellulose are complex and low yielding (Howard et al. 2003). Therefore, the cultivation of edible mushrooms is a crucial method for the bioconversion of many types of low-value lignocellulosic wastes (Choudhary et al. 2009).

In the present study, the activities of extracellular enzymes, such as carboxymethylcellulase (CMCase) and laccase, and the cellulose degradation rates of three kinds of edible fungi, namely, *Coprinus comatus*, *Agaricus brunnescens* Peck and *Pleurotus ostreatus*, were investigated under different culture conditions. Suitable strain and appropriate culture cycle were also determined by analyzing the trend of changes in the extracellular enzyme activities of edible fungi that could be used as biological agents for the organic recycling of agricultural wastes.

## Materials and methods

### Microorganisms

Three strains of edible fungi, namely, *C. comatus*, *A. brunnescens* and *P. ostreatus*, were obtained from technology research & development center for edible fungus of Tianjin agricultural university, and maintained on Potato Dextrose Agar (PDA) solid medium at 25 °C.

### Culture conditions and preparation of enzyme source

Until the mycelium was full, liquid-state fermentation was carried out in 500 mL conical flasks containing 100 mL of liquid fermentation medium (5 g/L amylum solani, 20 g/L glucose, 10 g/L straw, 2 g/LKH_2_PO_4_, 1 g/LMgSO_4_, 0.1 g/L CuSO_4_, and 2 g/L peptone at pH 4–6). Water in the *A. brunnescens* medium was composed of the filtrate of 30% peat soil boiled for 30 min. The edible fungi were cultured at 25 °C and shaken at 120 rpm for 10 days. On day 3, 2 mL of each sample was extracted and centrifuged at 4000 rpm for 20 min. The supernatant was obtained for enzyme activity determination.

Solid-state fermentation was conducted in a wheat grain medium containing 92%–93% wheat, 5% bran, and 2% gypsum. In the *C. comatus* and *A. brunnescens* culture media, 1% cow dung was added. In the *A. brunnescens* medium, water was composed of the filtrate of 30% peat soil boiled for 30 min. The edible fungi were cultured at 25 °C for 15–20 days. When the mycelium grew to 25%, 50%, 75%, and 100% of the culture bottle, 5 g of the sample was ground and transferred into 20 mL of water and extracted at 30 °C for 2 h. The solution was filtered and used for enzyme activity determination.

### CMCase and xylanase activity assay

CMCase and xylanase activities were determined in accordance with previously described methods (Wood and Bhat 1988) with some modifications. In brief, 0.5 mL of appropriately diluted enzyme was extracted with 1.5 mL of 1% CMC citric acid buffer (1% beech xylanase buffer) at pH 5.0 and 40 °C for 30 min. The reaction was terminated by adding 3 mL of 3,5-dinitrosalicylic acid reagent and boiled for 15 min. Then, 1 mL of 40% sodium potassium tartrate was added, and absorbance was measured at 540 nm against the blank (without enzyme filtrate). One unit of CMCase activity was expressed as 1 µmol of glucose/xylose liberated from each milliliter of enzyme per minute under assay conditions.

### Laccase activity assay

The laccase activity was determined spectrophotometrically at 436 nm (ε436 = 29,300 M^−1^ cm^−1^) for 5 min by using ABTS as a colorimetric substrate and an assay mixture containing 1 mM ABTS in 0.2 M sodium citrate buffer at pH 4.0 and incubated for 15 min at 30 °C before the measurement was performed. One activity unit (U) was defined as the amount of enzyme that oxidized 1 µmol of ABTS per minute at 25 °C, and the activities were expressed in units per liter.

### Cellulose content determination

Before and after fermentation was performed, 1 g of the samples was added to 100 mL of neutral detergent at 100 °C for 30 min. The filtrate was washed with 95% ethanol, anhydrous ethanol, and acetone until clear and then oven dried.

The residue was added to 2 mol/L hydrochloric acid solution at 100 °C for 30 min. The filtrate was washed with 95% ethanol, anhydrous ethanol, and acetone until clear and oven dried. The residue quality was weighed as W_1_.

The residue was added to precooled 72% sulfuric acid and degraded for 4 h at room temperature. Distilled water was then added to wash the residue to pH 6.5, and the dry residue quality was weighed as W_2_. The content of cellulose was W_1_ − W_2_.

### Data statistics

Comparisons between groups were performed by one-way analysis of variance followed by Bonferroni post hoc test (SPSS software package version 17.0, SPSS Inc., Chicago, IL, USA). The level of significance was set at P < 0.05.

## Results

### Intracellular enzyme activity of *C. comatus*

**Fig. 1:**
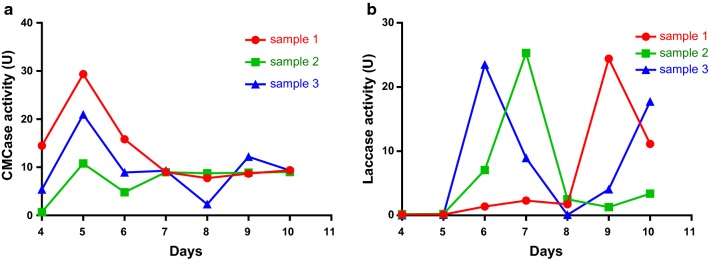
**a** CMCase activity of *C. comatus* during liquid-state fermentation. **b** Laccase activity of *C. comatus* during liquid-state fermentation

We compared the intracellular enzyme activities of *C. comatus*, *A. brunnescens*, and *P. ostreatus* in liquid-and solid-state fermentation. The results showed that the CMCase activity of *C. comatus* reached the maximum on day 5 during the liquid-state fermentation. The highest enzyme activity was 29.3538 U, and the enzyme activity plateaued on days 6 and 7 (Fig. 1a). The laccase activity of *C. comatus* reached its maximum value on day 7. The highest enzyme activity was 25.3129 U, and the enzyme activity remained low on the remaining days (Fig. 1b).

**Fig. 2:**
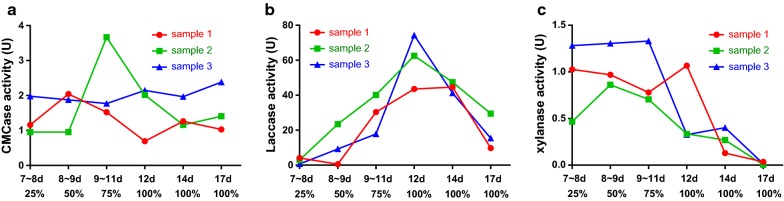
**a** CMCase activity of *C. comatus* during solid-state fermentation. **b** Laccase activity of *C. comatus* during solid-state fermentation. **c** Xylanase activity of *C. comatus* during solid-state fermentation

Figure 2a shows that the CMCase activity of *C. comatus* remained low during solid-state fermentation, and one of the samples reached its maximum value on days 9–11. The mycelial area reached 75%, and the enzyme activity was 3.666 U. The laccase activity of *C. comatus* continuously increased and peaked at 62.5427 U on day 12 (Fig. 2b). The xylanase activity of *C. comatus* was high at the early stage of fermentation, and the highest enzyme activity was 1.3306 U, which began to decrease after 12 days (Fig. 2c).

### Intracellular enzyme activity of *A. brunnescens*

**Fig. 3:**
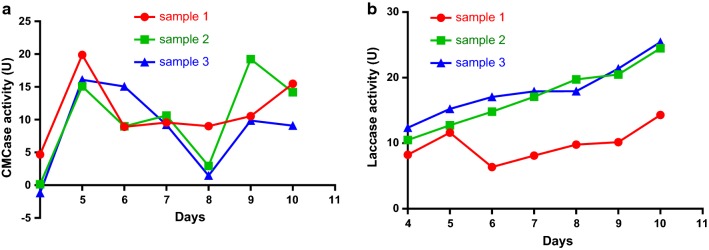
**a** CMCase activity of *A. brunnescens* during liquid-state fermentation. **b** Laccase activity of *A. brunnescens* during liquid-state fermentation

We compared the intracellular enzyme activity of *A. brunnescens* under different fermentation conditions by detecting the activities of CMCase, laccase, and xylanase. Figure 3a shows that the CMCase activity of *A. brunnescens* peaked on day 5 (19.8819 U) during liquid-state fermentation, and the activity increased slightly on days 9 and 10. The laccase activity of *A. brunnescens* increased continuously during fermentation and reached 25.4266 U on day 10 (Fig. 3b).

**Fig. 4:**
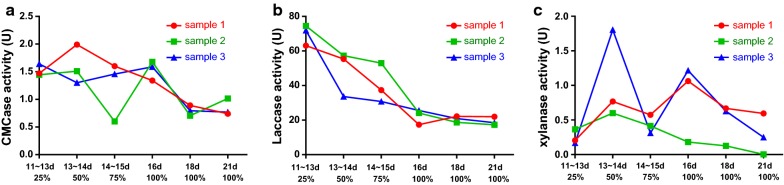
**a** CMCase activity of *A. brunnescens* during solid-state fermentation. **b** Laccase activity of *A. brunnescens* during solid-state fermentation. **c** Xylanase activity of *A. brunnescens* during solid-state fermentation

During solid-state fermentation, the CMCase activity of *A. brunnescens* was high at the initial stage of fermentation. The highest enzyme activity was up to 1.9905 U, and the enzyme activity decreased slightly during fermentation (Fig. 4a). In Fig. 4b, the laccase activity of *A. brunnescens* reached the highest level (74.5165 U) on days 11–13 but continuously decreased at the late stage. The xylanase activity of *A. brunnescens* was high on days 13, 14, and 16, and the highest was 1.8035 U (Fig. 4c).

### Intracellular enzyme activity of *P. ostreatus*

**Fig. 5:**
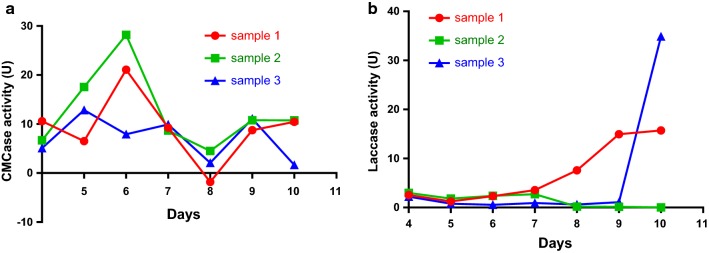
**a** CMCase activity of *P. ostreatus* during liquid-state fermentation. **b** Laccase activity of *P. ostreatus* during liquid-state fermentation

The activity of the intracellular enzymes of *P. ostreatus* was determined under solid-and liquid-state fermentation. During liquid-state fermentation, the activity of CMCase peaked on day 6. The highest enzyme activity was 28.1975 U, and the activity increased slightly on days 9 and 10 (Fig. 5a). Figure 5b shows that the laccase activity remained low on the first 7 days and increased to 34.8692 U on day 10.

**Fig. 6:**
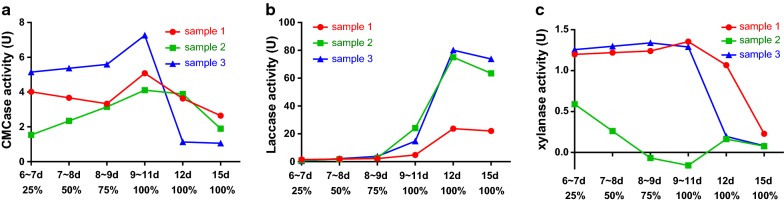
**a** CMCase activity of *P. ostreatus* during solid-state fermentation. **b** Laccase activity of *P. ostreatus* during solid-state fermentation. **c** Xylanase activity of *P. ostreatus* during solid-state fermentation

The CMCase activity of *P. ostreatus* increased slowly at the early stage of solid-state fermentation and peaked at 7.2657 U on days 11–13 (Fig. 6a). The laccase activity of *P. ostreatus* began to increase gradually on days 8 and 9, peaked at 80.1479 U on day 12, and decreased slowly (Fig. 6b). Figure 5a illustrates that the xylanase activity remained high before days 9–11 (1.3553 U) and began to decrease after 11 days.

### Cellulose degradation rates of *C. comatus*, *A. brunnescens*, and *P. ostreatus*

We analyzed the data and obtained the degradation rate of cellulose (Table 1) by comparing the cellulose contents of the three edible fungi before and after fermentation. The results showed that the degradation rates of cellulose were as follows: *C. comatus*, 25.24%; *A. brunnescens*, 37.75%; and *P. ostreatus*, 21.05%.

**Table 1:** Cellulose degradation rate of three kinds of edible fungi

	Before fermentation (%)	After fermentation		Cellulose degradation rate (%)
*Coprinus comatus*	28.64	1	21.02%	25.24
		2	20.68%	
		3	22.53%	
		Average	21.41%	
*Agaricus brunnescens Peck*	25.03	1	13.95%	37.75
		2	14.86%	
		3	17.94%	
		Average	15.58%	
*Pleurotus ostreatus*	28.64	1	25.37%	21.05
		2	21.79%	
		3	20.67%	
		Average	22.61%	

## Discussion

Crop straw is a rich renewable resource, but it causes environmental pollution that is considered a major problem in modern agriculture (Novaes et al. 2010; Sanderson 2011). Lignocellulose, which is abundant in crop straw, is the main component limiting straw degradation (Ćilerdžić et al. 2017). Laccase and xylanase are the main components of lignocellulase. Microbial strains, such as fungi, can effectively degrade lignocellulose by secreting lignocellulosin-degrading enzymes through the oxidative cleavage of chemical bonds (Liew et al. 2011; Yang et al. 2011; Zeng et al. 2006). Edible fungi have become a new field in the development of natural drug resources because of their special biological effects and medicinal values (Chihara et al. 1970; Petrovai and Diana 1970). Therefore, determining the appropriate culture conditions for edible fungi can alleviate the difficulty in degrading lignocellulose and promote the growth of edible fungi.

In the present study, the extracellular enzyme levels of three edible fungi, namely, *C. comatus*, *A. brunnescens*, and *P. ostreatus* were determined under different culture conditions. The results showed that the enzyme activity in liquid-state fermentation was better than that in solid-state fermentation. The enzyme activity peaked on days 5 and 6, and the peak activity of *C. comatus* could reach 29.3538 U. For the laccase activity, solid-state fermentation was better than liquid-state fermentation, and the enzyme activity peaked on days 8–12. The highest laccase activity of *P. ostreatus* was 80.1479 U. Furthermore, the highest xylanase activity of *A. brunnescens* was 1.8035 U observed on days 13–14. The degradation rate of cellulose was the highest (21.05%). This study analyzed the activities of extracellular enzymes of edible fungi under different conditions to understand the pattern of the extracellular enzyme secretion of edible fungi, to determine the enhanced culture conditions, and to improve the yield of the three kinds of edible fungi. Increasing the secretion of lignocellulase can maximize edible fungi to degrade crop straw. Further studies on nutrient components and combinations can be performed to optimize the enzyme production levels and degradation ability of crop stalks.
